# Deformable abdominal phantom for the validation of real‐time image guidance and deformable dose accumulation

**DOI:** 10.1002/acm2.12687

**Published:** 2019-07-29

**Authors:** Charles K. Matrosic, Jennifer Hull, Benjamin Palmer, Wesley Culberson, Bryan Bednarz

**Affiliations:** ^1^ School of Medicine and Public Health, Department of Medical Physics University of Wisconsin‐Madison Madison Wisconsin USA

**Keywords:** Deformable dose accumulation, motion management, phantoms, three‐dimensional dosimetry

## Abstract

**Purpose:**

End‐to‐end testing with quality assurance (QA) phantoms for deformable dose accumulation and real‐time image‐guided radiotherapy (IGRT) has recently been recommended by American Association of Physicists in Medicine (AAPM) Task Groups 132 and 76. The goal of this work was to develop a deformable abdominal phantom containing a deformable three‐dimensional dosimeter that could provide robust testing of these systems.

**Methods:**

The deformable abdominal phantom was fabricated from polyvinyl chloride plastisol and phantom motion was simulated with a programmable motion stage and plunger. A deformable normoxic polyacrylamide gel (nPAG) dosimeter was incorporated into the phantom apparatus to represent a liver tumor. Dosimeter data were acquired using magnetic resonance imaging (MRI). Static measurements were compared to planned dose distributions. Static and dynamic deformations were used to simulate inter‐ and intrafractional motion in the phantom and measurements were compared to baseline measurements.

**Results:**

The statically irradiated dosimeters matched the planned dose distribution with an average γ pass rates of 97.0 ± 0.5% and 97.5 ± 0.2% for 3%/5 mm and 5%/5 mm criteria, respectively. Static deformations caused measured dose distribution shifts toward the phantom plunger. During the dynamic deformation experiment, the dosimeter that utilized beam gating showed an improvement in the γ pass rate compared to the dosimeter that did not.

**Conclusions:**

A deformable abdominal phantom apparatus which incorporates a deformable nPAG dosimeter was developed to test real‐time IGRT systems and deformable dose accumulation algorithms. This apparatus was used to benchmark simple static irradiations in which it was found that measurements match well to the planned distributions. Deformable dose accumulation could be tested by directly measuring the shifts and blurring of the target dose due to interfractional organ deformation and motion. Dosimetric improvements were achieved from the motion management during intrafractional motion.

## 1 INTRODUCTION

Patient motion can reduce the precision of external beam radiotherapy (EBRT), resulting in decreased target coverage and irradiation of nearby healthy structures. This motion can be especially detrimental in the thoracic and abdominal regions of the body, where translational motion and deformation can be on the order of several centimeters.^1^, ^2^, ^3^, ^4^ Image‐guided radiation therapy (IGRT) has improved the precision of radiotherapy by using different imaging systems to reduce interfractional and intrafractional motion uncertainties. Interfractional motion is monitored during treatment using daily patient imaging data and deformable dose accumulation algorithms. By applying deformable image registration (DIR), the dose each day is deformed to the original patient imaging data that were used to plan the initial treatment.^5^ Daily dose maps are used to estimate the delivered cumulative dose distribution during the course of the treatment and assist clinicians in making informed decisions about the possible adaptations to a patient treatment course.^6^, ^7^ Furthermore, real‐time motion management techniques have been developed to account for intrafractional motion during each fraction resulting from respiratory motion, cardiac motion, peristalsis, etc. During delivery, the treatment is adapted to account for the motion of the target by either gating or tracking the treatment beam.^8^, ^9^ Numerous imaging modalities have been utilized to monitor target motion including optical surface tracking,^10^, ^11^ magnetic resonance imaging (MRI) guidance,^12^, ^13^ ultrasound guidance,^14^, ^15^, ^16^ and fluoroscopy.^17^, ^18^ Both interfractional and intrafractional motion management strategies have led to the reduction of treatment margins used for a variety of tumor types seen clinically.^19^

Clinical implementation of motion management systems requires that they first be validated through measurement. American Association of Physicists in Medicine Task Groups 76 and 132, which discuss respiratory motion management and image registration algorithms in radiotherapy, both recommend end‐to‐end testing of these systems using quality assurance (QA) phantoms.^20^, ^21^ Robust testing of these systems requires a phantom that is able to simulate translational motion and deformation, be compatible with a variety of imaging modalities, and be reusable for multiple experiments. Ideally, these phantoms would also provide three‐dimensional (3D) dosimetry, and steps toward the clinical implementation of deformable 3D dosimetry have been made. DEFGEL is a deformable normoxic polyacrylamide gel (nPAG) dosimeter contained within a latex membrane initially proposed Yeo et al. that has been proven by previous work to be well suited for 3D deformable dosimetry.^22^ The dosimeter can be irradiated in a deformed state and read out in its original, undeformed state, allowing for the comparison of deformed dose calculations by deformable dose accumulation algorithms to physical measurement. Furthermore, this deformability allows for realistic dynamic deformation of a target during the test of real‐time IGRT systems.^23^ Other deformable dosimeters have been developed specifically for the testing of deformable dose accumulation algorithms, some examples being Presage‐Def, FlexyDos3D, and the incorporation of normoxic methacrylic acid gel (nMAG) in low‐density polyethylene containers (LDPE).^24^, ^25^, ^26^ However, none of these dosimeters have been incorporated into fully deformable phantoms.

To the best of our knowledge, no phantom exists that includes all of these aforementioned features required to adequately provide robust testing of IGRT systems. For example, some commercial phantoms incorporate rigid motion during treatment but do not to mimic both the translational motion and deformation of the human body. This is especially crucial in testing deformable dose accumulation algorithms since both the rigid image registration and DIR of an algorithm must be validated. Additionally, commercial motion phantoms are limited in the scope of compatible imaging modalities. The purpose of this work was to develop a deformable anthropomorphic phantom that incorporates deformable 3D dosimetry and is compatible with a variety of imaging modalities including both MRI and ultrasound. This phantom and dosimeter pairing was tested for its ability to perform the static deformation measurements beneficial for future testing of deformable dose accumulation algorithms and the dynamic deformation measurements required for future testing of IGRT motion management systems.

## 2 MATERIALS AND METHODS

### 2.A Deformable abdominal phantom development

**Figure 1:**
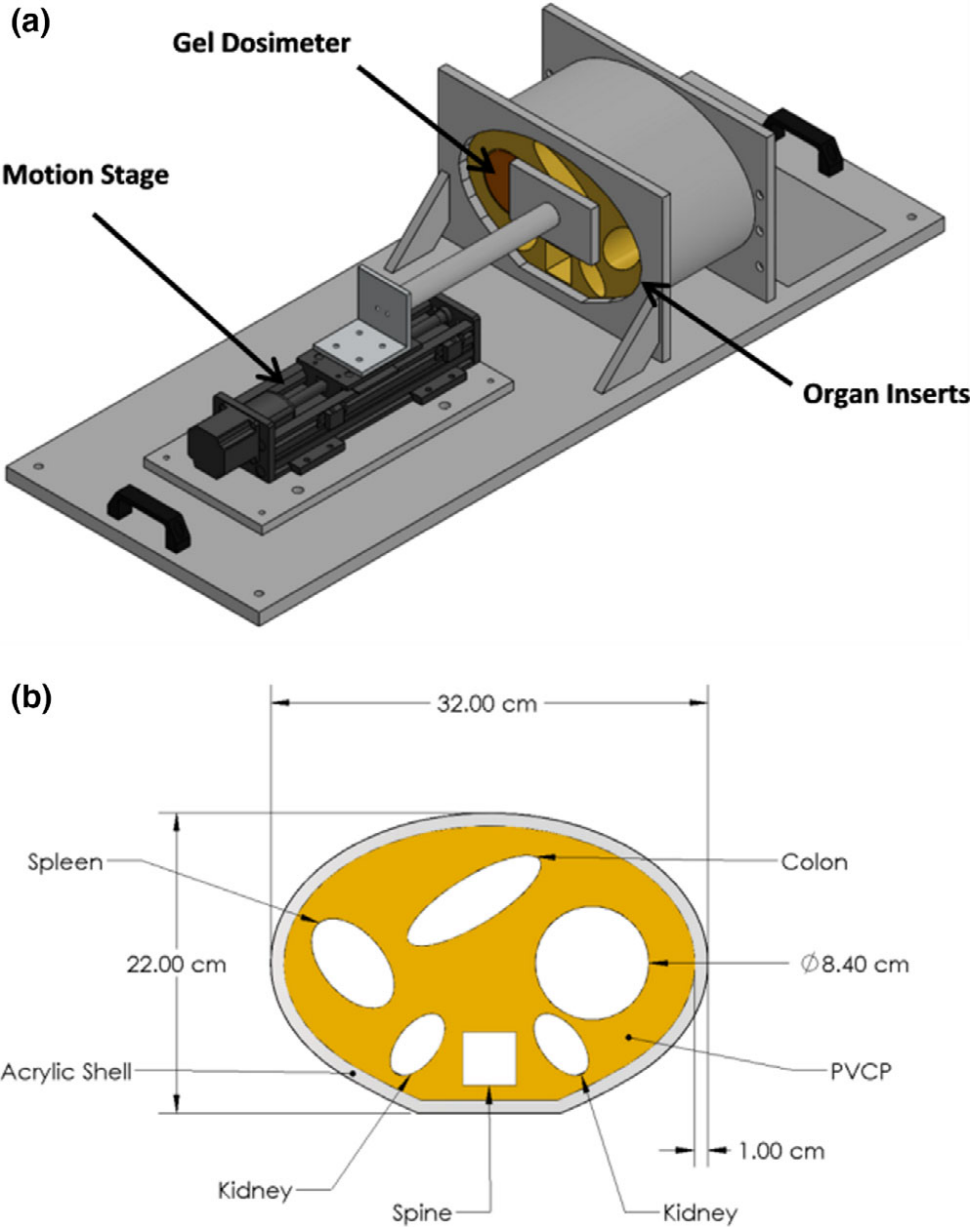
a) Rendering and b) axial cross section of the deformable abdominal phantom that was developed. The phantom platform features a programmable motion stage and plunger apparatus to drive motion, a deformable phantom section with low‐density organ sections, and a cavity for containing a removable deformable 3D dosimeter. 3D, three‐dimensional.

The phantom apparatus illustrated in Fig. 1 features a deformable plastic abdominal phantom housed in an acrylic shell mounted on an acrylic board. The phantom motion and deformation is driven by a programmable motion stage and plunger incident upon the abdominal section of the apparatus highlighted in yellow in Fig. 1. The acrylic shell is open on both the cranial and caudal ends, allowing the piston to push the phantom and the phantom can both deform and deflect out the cranial end of the shell. The material used to fabricate the phantom was polyvinyl chloride plastisol (PVCP) (M‐F Manufacturing, Fort Worth, TX). PVCP has been used in previously published work as a material for developing deformable multimodal anthropomorphic phantoms, and has been shown to have mechanical properties similar to those of porcine abdominal organs.^27^, ^28^, ^29^, ^30^ During the heating of the liquid PVCP, additives were used to change the physical properties of the material. The addition of a hardener or softener increased or decreased the density of the PVCP, which modified the computed tomography (CT) and MRI properties of the material.^28^ This technique was utilized to create imaging contrast between different PVCP sections of a phantom.

To calibrate the relationship between the HU values of the different mixtures of regular plastisol with the percentage by volume of hardener or softener in the mixture, a set of 13 small cylindrical samples of PVCP mixtures was fabricated. The range of hardener percentages ranged from 0% to 100%, while the range of softener ranged from 0% to 45%, which was the highest percentage that could be fabricated without the sample losing its shape. The samples were scanned with a Siemens SOMATOM Definition Edge CT scanner (Siemens Healthcare, Forchheim, Germany), and Fiji^31^ was used to assess the mean HU value and the standard deviation of this mean value within a region of interest (ROI) for each sample. The resultant PVCP HU calibration fit for the addition of hardener was:

$$HU=0.4237\left(P_{\mathrm{hard}}\right)+21.531$$

$$R^{2}=0.9476$$

and the PVCP HU calibration fit for the addition of softener was:

$$HU=-1.9063\left(P_{\mathrm{soft}}\right)+31.703$$

$$R^{2}=0.9681$$

where P_hard_ and P_soft_ were the percentage by volume of hardener and softener added to the mixture added, respectively.

**Figure 2:**
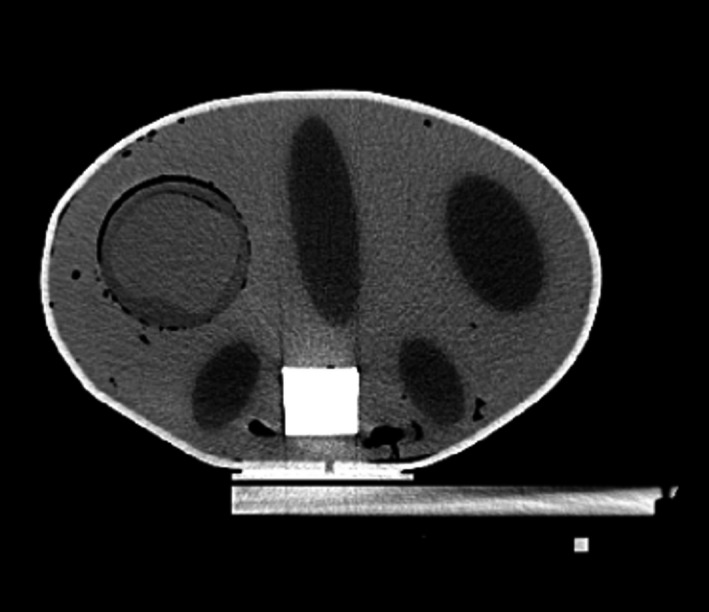
Axial slice of a CT image of the PVCP abdominal phantom. PVCP, polyvinyl chloride plastisol.

Two PVCP phantoms were fabricated for the following experiments, a lower contrast initial version and a higher contrast second phantom to improve contouring ease. The PVCP section of each phantom was fabricated by first pouring the bulk background PVCP to make up the majority of the phantom, while 3D‐printed inserts were left in place to create cavities for the organ and target sections of the phantom. The PVCP mixture used to make the background of the first phantom was regular PVCP with no additives, with an HU of approximately 27 HU, and the second phantom used 50% hardener by volume, resulting in an HU of approximately 43 HU. After this section had cooled, the 3D‐printed inserts were removed and the sections representing the kidneys, spleen, and duodenum were filled with a lower density PVCP. In the case of the first phantom used 20% softener by volume, resulting in an HU of approximately −6 HU, while the second used 35% softener by volume resulting in an HU of −35 HU. The section representing the spine was filled with a high density casting material (Perfect Cast Casting Material, Skullduggery Inc, Anaheim, CA). An additional open cavity near the liver region was created to contain a removable deformable 3D dosimeter to represent a liver tumor, which will be discussed further in following sections. An example axial slice of a CT of the second phantom is shown in Fig. 2.

The programmable motion stage utilized in this apparatus was the surrogate‐axis motion stage of the Washington University 4D Phantom.^32^ The motion stage was fully programmable and can be used to either hold static positions or to dynamically move with a respiratory motion trace during treatment, with a positioning precision within approximately 10 microns. A piston was attached to the motion stage to contact the center of the caudal end of the PVCP phantom section with a 4.5 inch by 3 inch rectangular block of acrylic. The pairing of the motion stage with the plunger resulted in a primarily cranial‐caudal deflection of the PVCP section of the phantom apparatus, with a maximum 2 cm deflection of the superior side of the phantom without any measurable damage to the phantom. The location of the plunger contact point and the acrylic shell surrounding the PVCP resulted in the majority of the deformation and deflection of the phantom to be within the medial sections of the phantom, while the lateral edges showed a lower magnitude of motion. While the motion stage used in this work was made of ferromagnetic materials, it could be easily removed from the phantom apparatus and replaced with an MR‐compatible motion‐driving system in the future.

### 2.B Deformable 3D dosimeter

**Figure 3:**
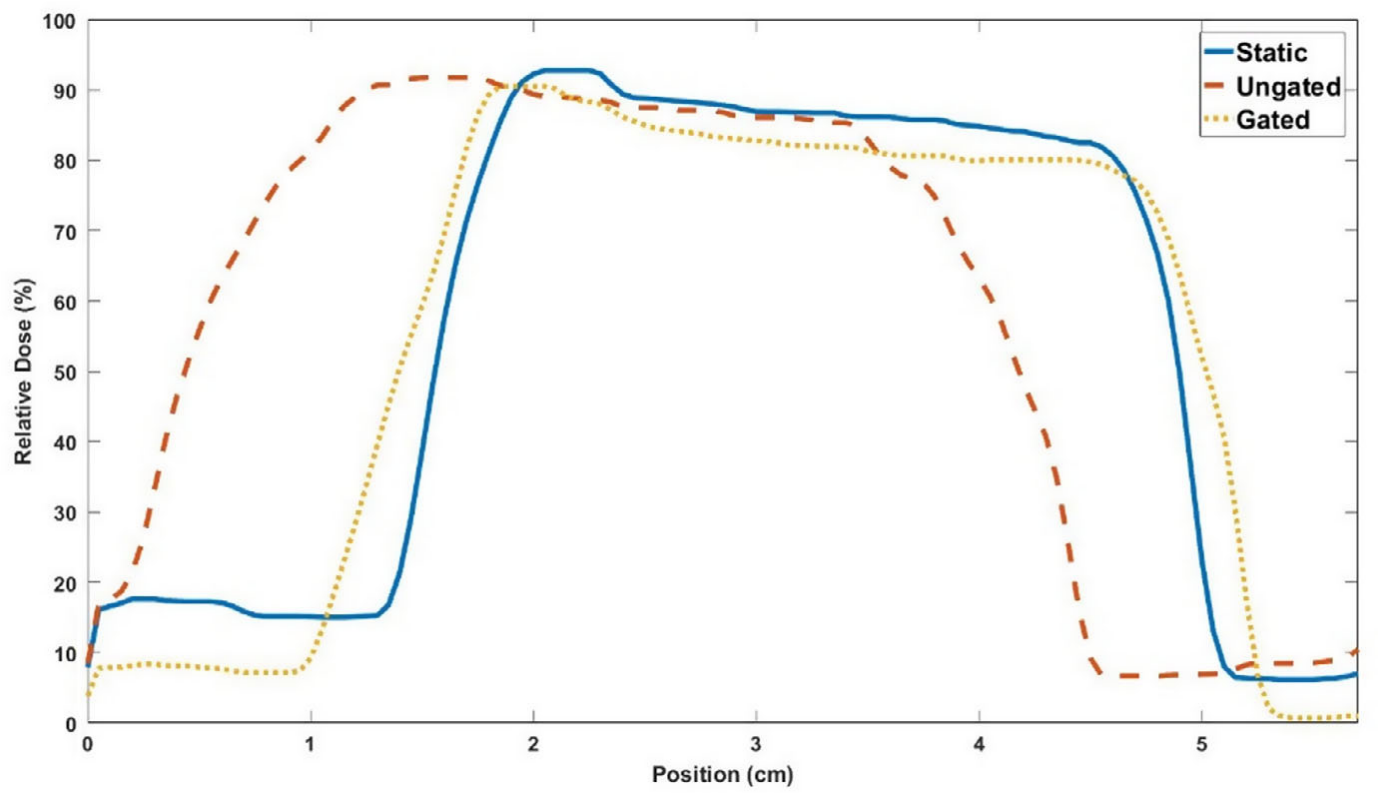
a) Model of the molding method used to fabricate the PVCP shells which encased the nPAG. b) Model of the 3D‐printed insert used to create the asymmetric inner cavities in the PVCP shells. c) Image of a completed deformable dosimeter. 3D, three‐dimensional; nPAG, normoxic polyacrylamide gel; PVCP, polyvinyl chloride plastisol.

A DEFGEL nPAG dosimeter encased in a PVCP shell was paired with the abdominal phantom. The DEFGEL was fabricated using the materials, heating methods, and mixing techniques described by Yeo et al.^22^ The gel was injected into deformable PVCP shells. The PVCP shells were fabricated using an acrylic outer ring and a 3D‐printed insert to create the shape of the inner cavity [Fig. 3(a)], which governs the shape of the nPAG dosimeter. An asymmetric inner cavity was created to better represent the asymmetry of a tumor volume [Fig. 3(b)]. The resultant gel dimensions were approximately 5 cm tall and 6.3 cm in diameter. The shells were capped and sealed with additional PVCP prior to injecting the nPAG through a hole in the top of the dosimeter which was also sealed with PVCP after injection. CT fiducial markers were placed in each PVCP shell for the purposes of image registration during data analysis. One fiducial marker was implanted inside the base of each shell and two were implanted inside each cap, resulting in clear, bright fiducials in CT and clear, dark cavities in an MR acquisition. An image of a completed dosimeter is shown in [Fig. 3(c)].

### 2.C Static irradiation test

**Figure 4:**
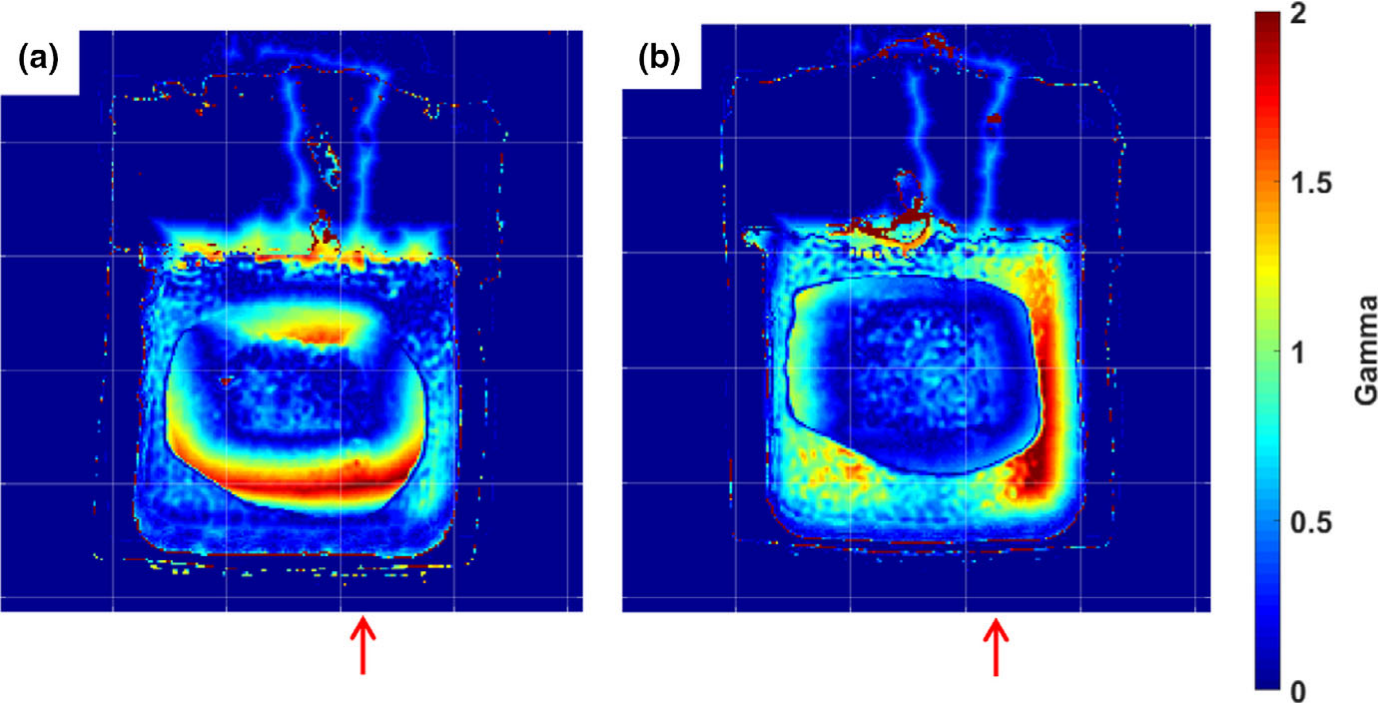
The irradiation setup of the abdominal phantom apparatus.

Measurements of static irradiation conditions using the deformable dosimeter in the first phantom were compared to the calculated dose distribution in Eclipse^TM^ Treatment Planning System (TPS) (Varian Medical Systems, Palo Alto, California). The purpose of the experiment was to test the ability of the apparatus to match planned dose distributions in cases without phantom motion or deformation. Four deformable nPAG dosimeters were created for this experiment, and each was imaged in phantom using CT. CT data were gathered using 120 kVp scans on a Siemens SOMATOM Definition Edge CT scanner. The dose response of the nPAG batch for the experiment was calibrated using one dosimeter in a four‐field treatment. This calibration treatment involved four 3 cm × 3 cm fields with varying weights, resulting in a 17 Gy target dose to the center of the dosimeter. A liver stereotatic body radiotherapy (SBRT) plan was optimized using a clinical protocol with target coverage of 95% by 99% of the 12 Gy prescription dose for a single fraction. In this experiment, the target region was defined as a 1.7 cm subtraction of the nPAG contour to avoid regions of oxygen inhibition. This resulted in a target volume which was approximately 2.3 cm tall and 3.5 cm in diameter. The dosimeters were irradiated using a Varian Clinac 21EX research linac with a 6 MV photon beam as shown in Fig. 4. Delivery QA of the liver SBRT treatment plan was assessed with a static gantry angle delivery to EBT3 film dosimeter (Ashland Inc., Bridgewater, NJ) placed at 10 cm depth within a Virtual Water™ (Med‐Cal Inc., Verona, WI) phantom, and resulted in a 98.0% γ pass rate for 3%/3 mm criteria.

After irradiation, the dosimeters were allowed to polymerize overnight and were imaged using MRI with a GE SIGNA^TM^ 3T PET/MRI (GE Healthcare, Waukesha, WI). A specialized 16‐echo multiple spin‐echo (MSE) pulse sequence was used to read out the nPAG dosimeters with a 40 ms echo spacing, an acquisition resolution of 1 mm × 1 mm × 3 mm, and a scan time of 44 min. These data were used to create R_2_ maps of each dosimeter by fitting the echoes to an exponential decay on a voxel‐by‐voxel basis. A calibration of R_2_ to dose was created with the TPS predicted dose distribution of the calibration dosimeter irradiated with the four‐field plan. The R_2_ map of the calibration dosimeter was coregistered to the TPS dose distribution using Amira^TM^ (ThermoFisher Scientific, Waltham, Massachusetts) to directly compare R_2_ values to their corresponding dose. The calibration fit used was a monoexponential fit described by Vandecasteele and DeDeene 33, 34 to capture the nonlinear nature of the dose response of the large range of doses, which ranged approximately 0–15 Gy. Dose maps measured by the deformable dosimeters were compared to their corresponding predicted dose distributions by Eclipse^TM^.

### 2.D Static deformation test

The effects of static deformation on the dose distributions were measured by the nPAG dosimeters in the second, higher contrast deformable phantom apparatus. The purpose of the investigation was to show the apparatus’ ability to measure the effects of static deformation for the purpose of testing deformable dose accumulation algorithms. Five dosimeters were created for this experiment and each was imaged with CT in phantom. One dosimeter was used to calibrate the nPAG batch dose response with a four‐field treatment plan similar to that of the static irradiation test, except with a maximum dose of 14 Gy, and a volumetric modulated arc therapy (VMAT) liver treatment plan was created for the remaining dosimeters. In this case, the treatment was scaled to a 3 Gy target dose, as opposed to the previous 12 Gy target dose to avoid dosimeter response saturation over multiple fraction deliveries. Delivery QA was performed using the same method as the static irradiation test, and resulted in a γ pass rate of 99.5% for 3%/3 mm criteria.

The first dosimeter had no additional deformation placed on it and was irradiated with a single treatment fraction serving as a baseline case. The second dosimeter underwent a centimeter of static deformation during a single treatment fraction irradiation, and the third dosimeter was deformed 2 cm during a single treatment fraction. The final dosimeter was irradiated with three fractions with the first fraction having no deformation, the second fraction having 1 cm of deformation, and the third fraction having 2 cm of deformation. This three‐fraction dosimeter was calibrated with the dose response of the calibration dosimeter irradiated with the four‐field plan, which covered a dose range from 0 Gy to 14 Gy. The single‐fraction dosimeters were calibrated using the dose response of the dosimeter that was left undeformed to ensure accurate calibration over the smaller 0–5 Gy dose range. During this experiment, the amount of deformation was quantified as the measured deflection by the phantom on the cranial edge due to the displacement of the motion stage plunger. The deflection of the cranial edge of the phantom was approximately half the magnitude of the displacement of the motion stage plunger on the caudal edge of the phantom, which implied that part of the force resulted translation of the phantom and part resulted in compression. After irradiation, each dosimeter was undeformed to its original shape. All dosimeters were MR imaged and analyzed after irradiation using the procedure described for the static irradiation benchmarking.

### 2.E Dynamic deformation test

The second phantom apparatus was used to study the effects of dynamic motion and deformation during irradiation and the influence of beam gating on a treatment delivery. During irradiation, the apparatus was set to dynamically move and deform while measuring dose for the testing of real‐time IGRT systems. Due to the gating limitations of the Clinac 21EX Linac research linac used for this work, the VMAT treatments that were originally planned to represent liver treatments were replanned and delivered as IMRT plans. Delivery QA was performed using the same method as the static irradiation test, and resulted in a γ pass rate of 100.0% for 3%/3 mm criteria. Three gel dosimeters were fabricated for this investigation following the aforementioned methods.

The use of motion and beam gating was varied to investigate their effects on treatment delivery. A test treatment was delivered to one of the dosimeters without any motion during the treatment and was used as a baseline and calibration case. A second dosimeter was deformed during the treatment in a sinusoidal pattern with a 1 cm peak deformation and period of 4 s without any motion compensation methods. It is important to note that the motion trace began with the dosimeter in the undeformed position, which was at the minimum of the sinusoid. The final dosimeter was deformed using the same motion trace as the second dosimeter, but the beam was gated to turn on when the dosimeter was in the undeformed position. The beam was gated using a customized gating switch described in the work of Shepard et al.^35^ using a beam‐on and beam‐off gating signal based on the known motion trace of the phantom apparatus. The beam was gated with a 30% duty cycle, which resulted in a 0.95 mm maximum residual plunger motion within the gating window, which equates to approximately 0.43 mm maximum residual motion of the cranial edge of the phantom.^36^

## 3 RESULTS

### 3.A Static irradiation test

**Figure 5:**
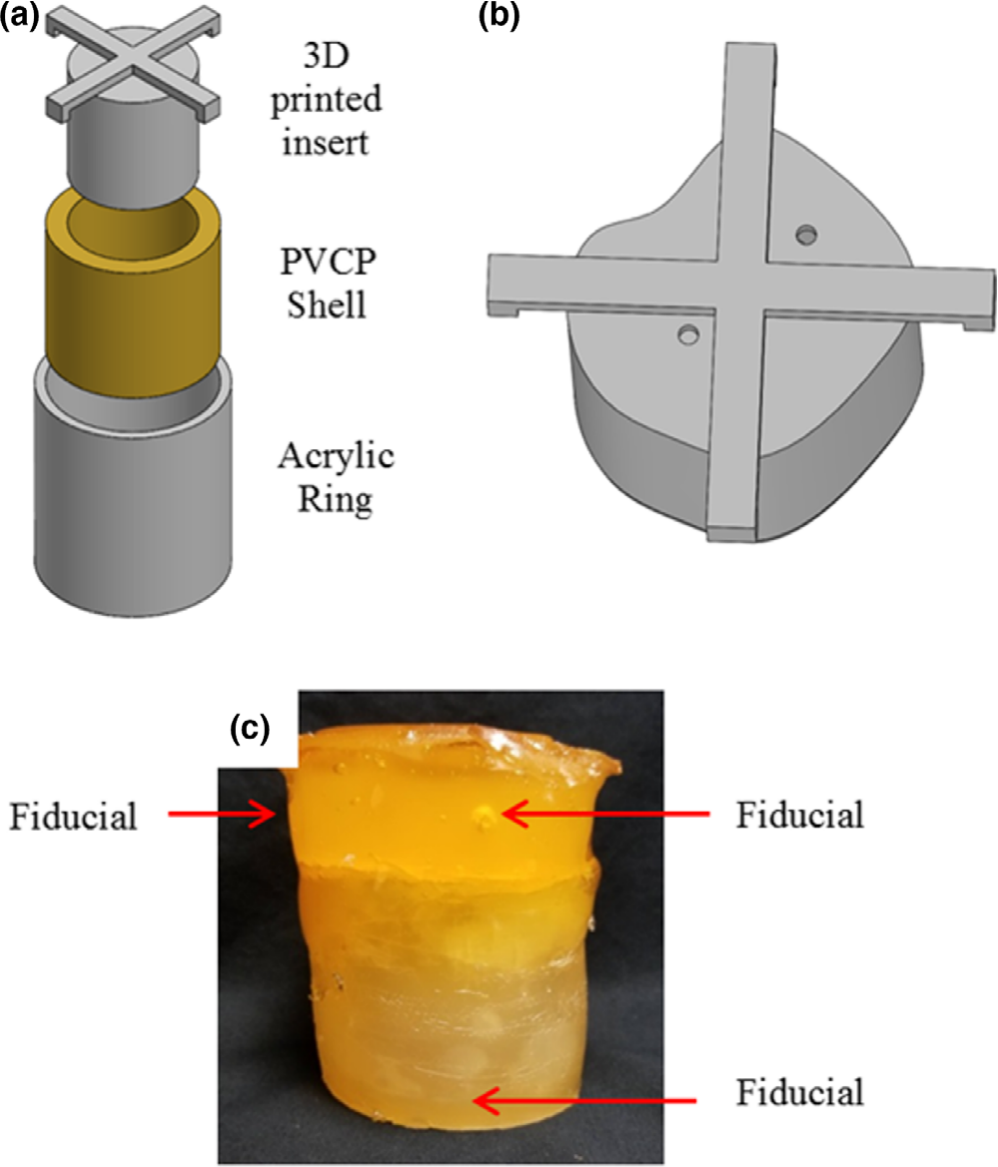
A central axial slice of the (a) nPAG measured dose distribution and (b) the TPS planned dose distribution for one of the three dosimeters irradiated with the liver SBRT treatment. The two distributions appear qualitatively similar in the high‐dose regions, but the nPAG distribution shows a distinct lack of response near the PVCP shell wall, which is shown in deep red. Each grid mark is spaced by 2.5 cm. nPAG, normoxic polyacrylamide gel; PVCP, polyvinyl chloride plastisol; SBRT, stereotatic body radiotherapy; TPS, treatment planning system.

**Figure 6:**
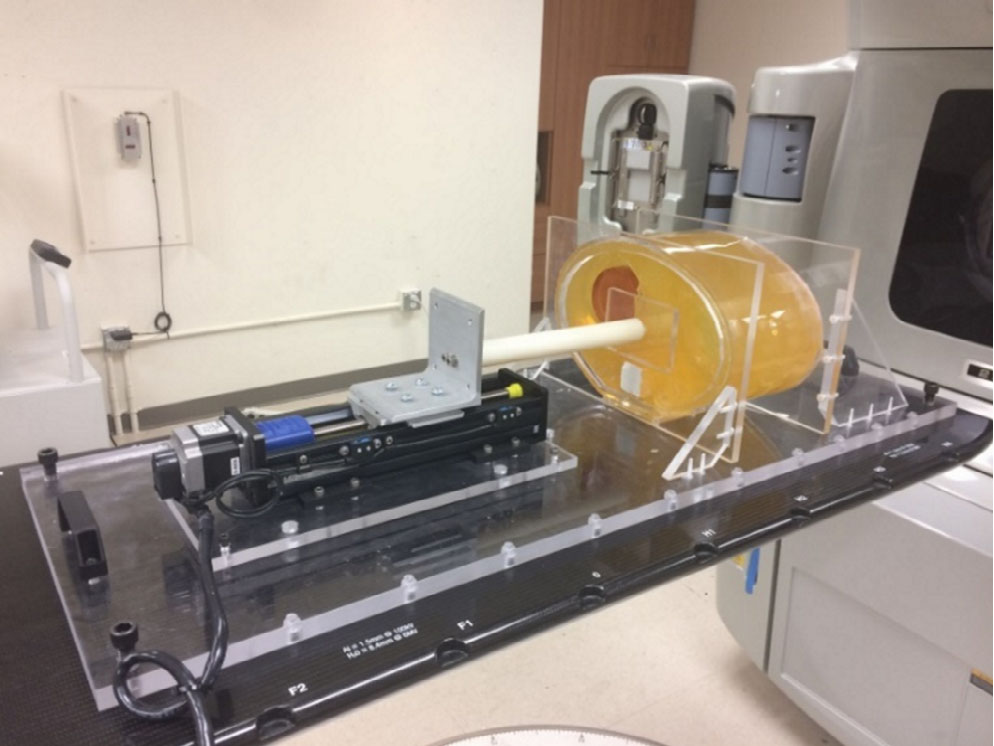
Central z‐profile of the dose distributions displayed in Fig. 5. The nPAG measured profile matched the TPS calculated profile in the high‐dose regions above 11 Gy with a 2.4% average absolute deviation. The nPAG showed a distinct falloff in dose due to oxygen inhibition near the edges of the gel due to oxygen inhibition. nPAG, normoxic polyacrylamide gel; TPS, treatment planning system.

An example of an axial slice of the planned dose distribution and measured distribution in one of the gel dosimeters is shown in Fig. 5. Qualitatively, the two dose distributions appear similar in the high‐dose target region of both distributions. A distinct drop‐off in measured dose can be seen near the edges of the gel dosimeter near the dark red PVCP shell. Profiles gathered through the middle of each dose distribution in the IEC defined z‐direction^37^ of the axial slices are shown in Fig. 6, illustrating that the dose distributions are similar in the high‐dose regions, but the edge of the dosimeter has a distinct falloff.

Each dosimeter irradiated with the liver SBRT plan was compared to its planned dose distribution using 3D γ‐analysis^38^, ^39^ with 3%/5 mm and 5%/5 mm criteria. The 5 mm distance‐to‐agreement (DTA) criterion was chosen so that the DTA criterion would be larger than the largest dimension of the dose map voxel size, 3 mm. The 5% dose difference was used to account for the 5% estimated k = 1 uncertainty of the gel dosimeter.^40^ Pass rates were calculated for the full volume and the central slice with a dose threshold of 20% of the maximum planned dose on both the measured and planned dose maps to remove low‐dose regions and regions of oxygen inhibition within the dosimeter and planned low‐dose regions. The average pass rates and standard deviations for each analysis are shown in Table 1.

**Table 1:** Average γ pass rates calculated for the three dosimeters irradiated with the liver SBRT treatment plan.

**Dose Difference (%)**	**DTA (mm)**	**Region**	**Pass Rate (%)**	**σ (%)**
3	5	Full Volume	97.0	0.5
5	5	Full Volume	97.5	0.2
3	5	Central Slice	99.7	0.5
5	5	Central Slice	100.0	0.0

SBRT, stereotatic body radiotherapy; DTA, distance‐to‐agreemen.

### 3.B Static deformation test

**Figure 7:**
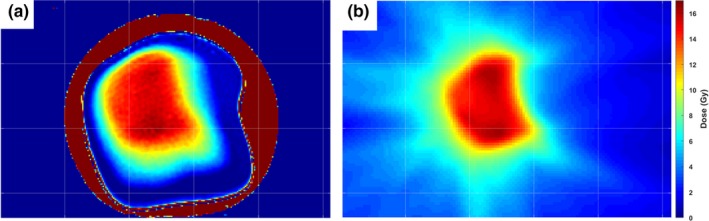
Central coronal slices of the isodose maps of the undeformed dosimeter (a), the dosimeter with 1 cm of deformation (b), the dosimeter with 2 cm of deformation (c), and the dosimeter that was irradiated with three fractions, each with a different deformation state (d). Red arrows approximate the location of the deformation site and the direction of deformation. Each grid mark is spaced by 2.5 cm.

**Figure 8:**
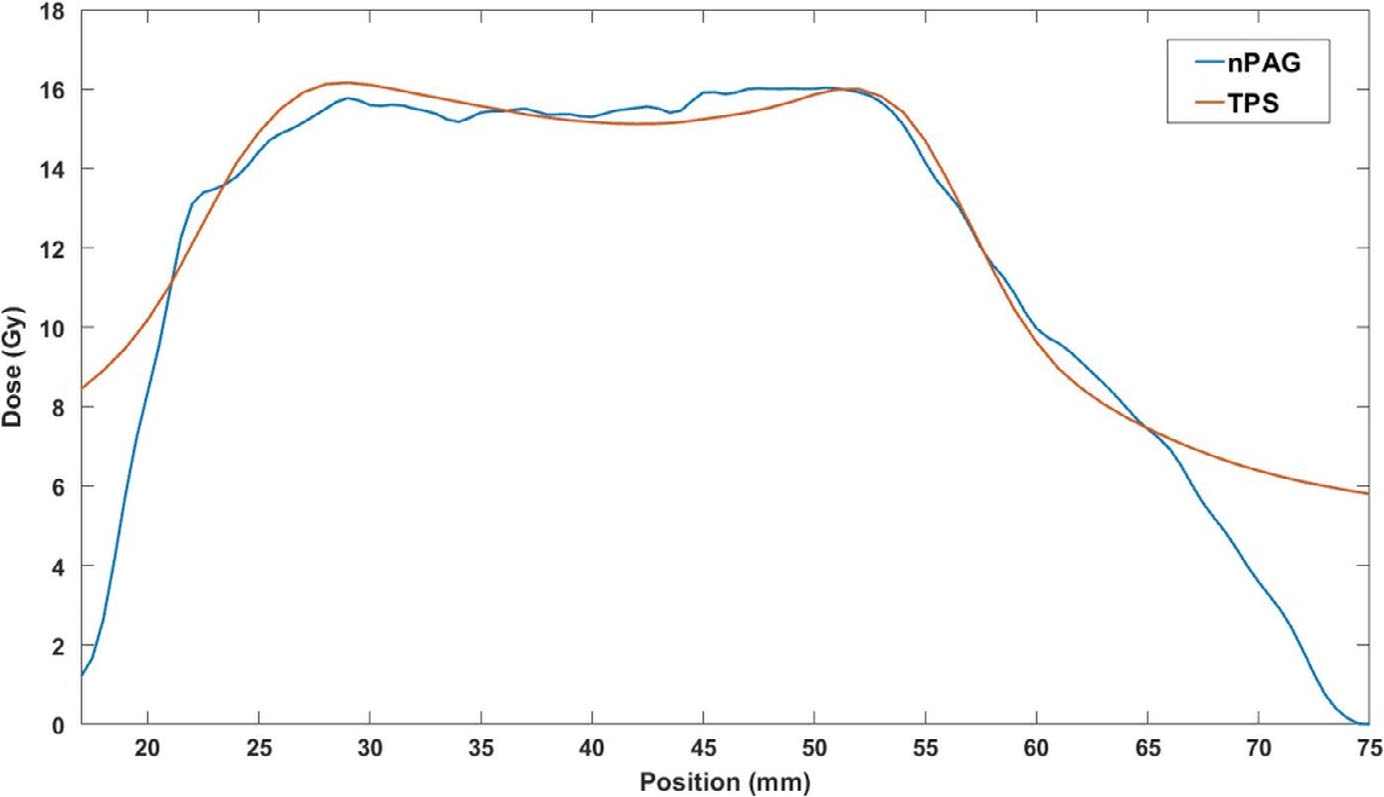
Central y‐profiles through each dose distribution measured by the dosimeters during the static deformation experiment.

Isodose plots of the central coronal slices of each measured dose distributions are shown in Fig. 7. Each target dose distribution was normalized to its maximum dose to allow an intercomparison of the different distributions. As more deformation was added to the dosimeters irradiated with single fractions, a larger shift in the isodose levels occurred. This shift was characterized by the elongation and displacement of dose distributions toward the site of deformation. In the case of Fig. 7, the deformation site was near the bottom‐right corner of each image. Note that the slightly smaller high‐dose region of high dose in [Fig. 7(c)] occurred due to the large deformation shifting the edge of the gel dosimeter into the beam, truncating the dose distribution, and resulting in some oxygen inhibition of polymerization. The dosimeter irradiated with three fractions and three different deformation states showed a blurring of dose over a larger area and a shift toward the site of deformation. Central profiles are shown for each dose map in the IEC defined y‐direction in Fig. 8. The single‐fraction deformed dosimeters showed distinct shifts relative to the undeformed dosimeter. The three‐fraction dosimeter showed a wider profile with a step‐like nature due to the three distinct deformation states during irradiation.

**Figure 9:**
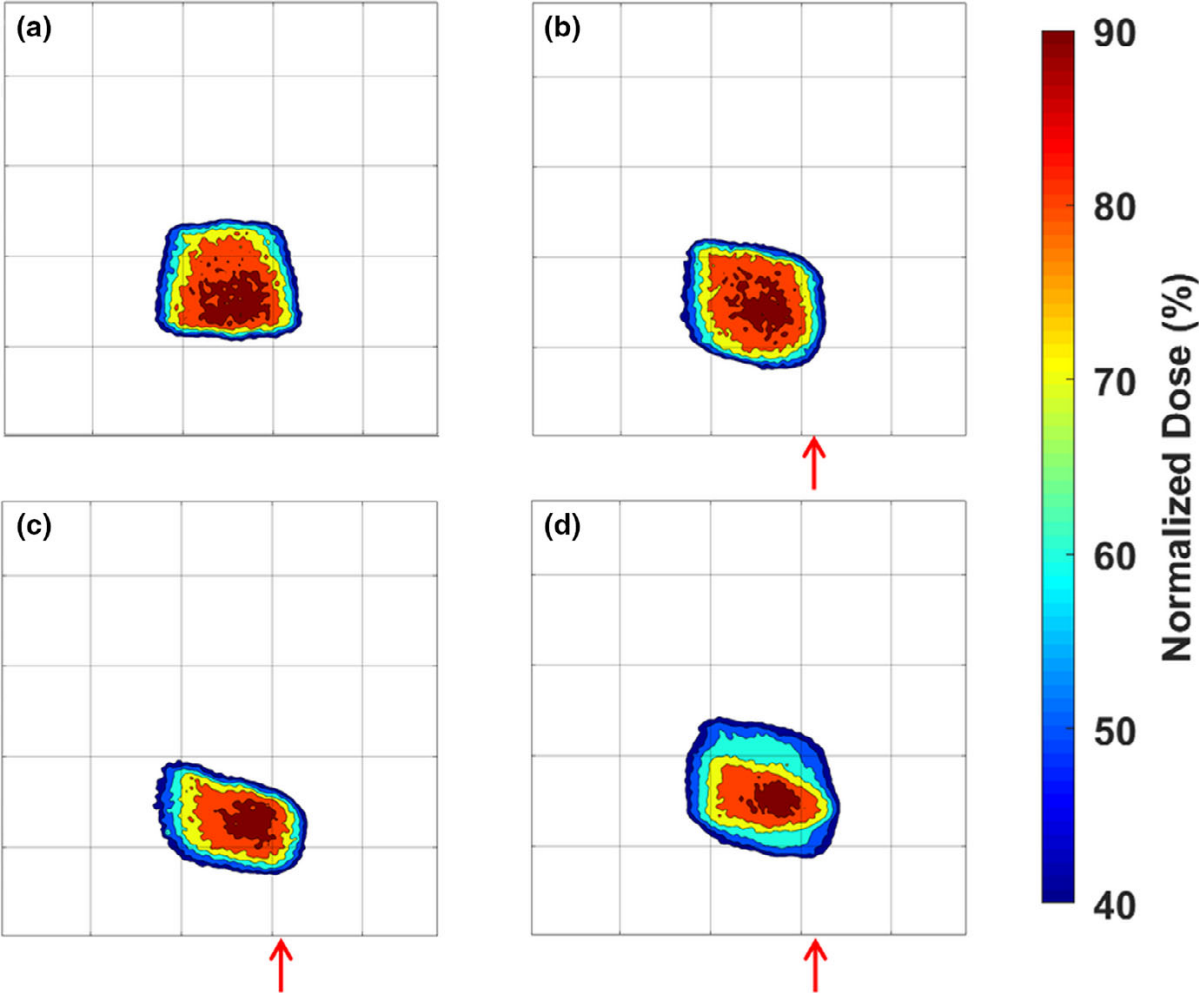
Central slices of γ maps utilizing 3%/5 mm criteria. The dosimeter that underwent 1 cm of deformation (a), 2 cm of deformation (b), and three fractions with the three deformation states (c) were compared to the baseline undeformed dosimeter for this analysis. Red arrows approximate the location of the deformation site and the direction of deformation. Each grid mark is spaced by 2.5 cm.

Each deformed dosimeter dose distribution was normalized to their maximum doses and compared to the static gel using 3%/5 mm and 5%/5 mm γ‐analysis criteria to quantify how similar each dosimeter was to the baseline undeformed case. Representative central coronal slices of the γ maps are shown in Fig. 9. As the amount of deformation increased for the single‐fraction dosimeters, larger bands of higher γ values appeared in the shifted sections of each dose map. In the three‐fraction irradiation case, blurring of the dose caused lower γ values in the higher dose regions than the single‐fraction dosimeters. Pass rates shown in Table 2 were calculated with a dose threshold meant to include points where either the baseline dose map or the deformed dosimeter dose map displays a dose over 20%.

**Table 2:** Pass γ rates of the comparison of the statically deformed dosimeters to the undeformed dosimeter.

**Dosimeter**	**Dose Difference (%)**	**DTA (mm)**	**Pass Rate (%)**
1 Fx, 1 cm deformation	3	5	91.0
1 Fx, 1 cm deformation	5	5	92.1
1 Fx, 2 cm deformation	3	5	82.0
1 Fx, 2 cm deformation	5	5	82.8
3 Fx	3	5	90.0
3 Fx	5	5	90.6

DTA, distance‐to‐agreemen.

### 3.C Dynamic deformation test

**Figure 10:**
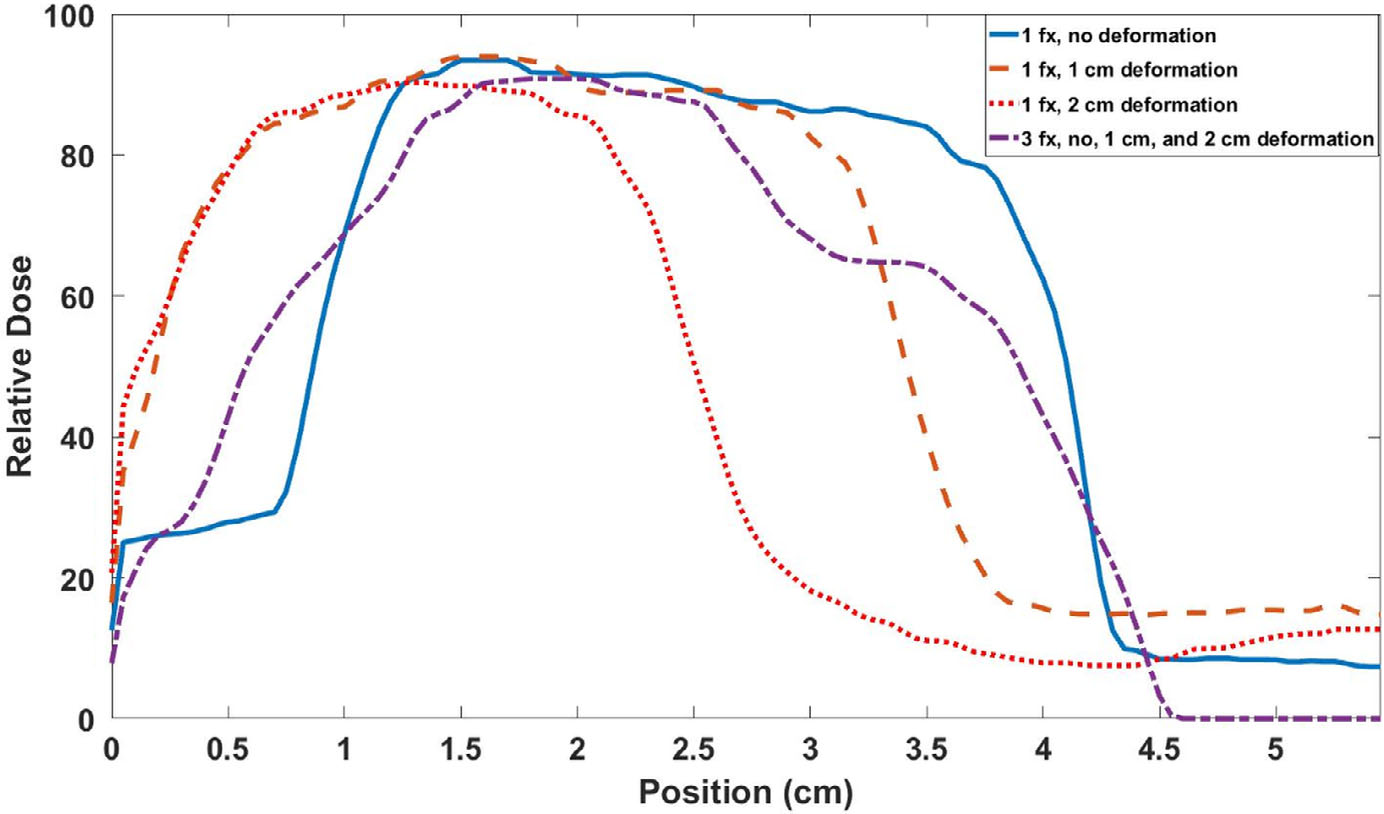
Central coronal slices of the isodose plots of the static undeformed dosimeter (a), the ungated dynamically deformed dosimeter (b), and the beam‐gated dynamically deformed dosimeter (c). Red arrows approximate the location of the deformation site and the direction of deformation. Each grid mark is spaced by 2.5 cm.

**Figure 11:**
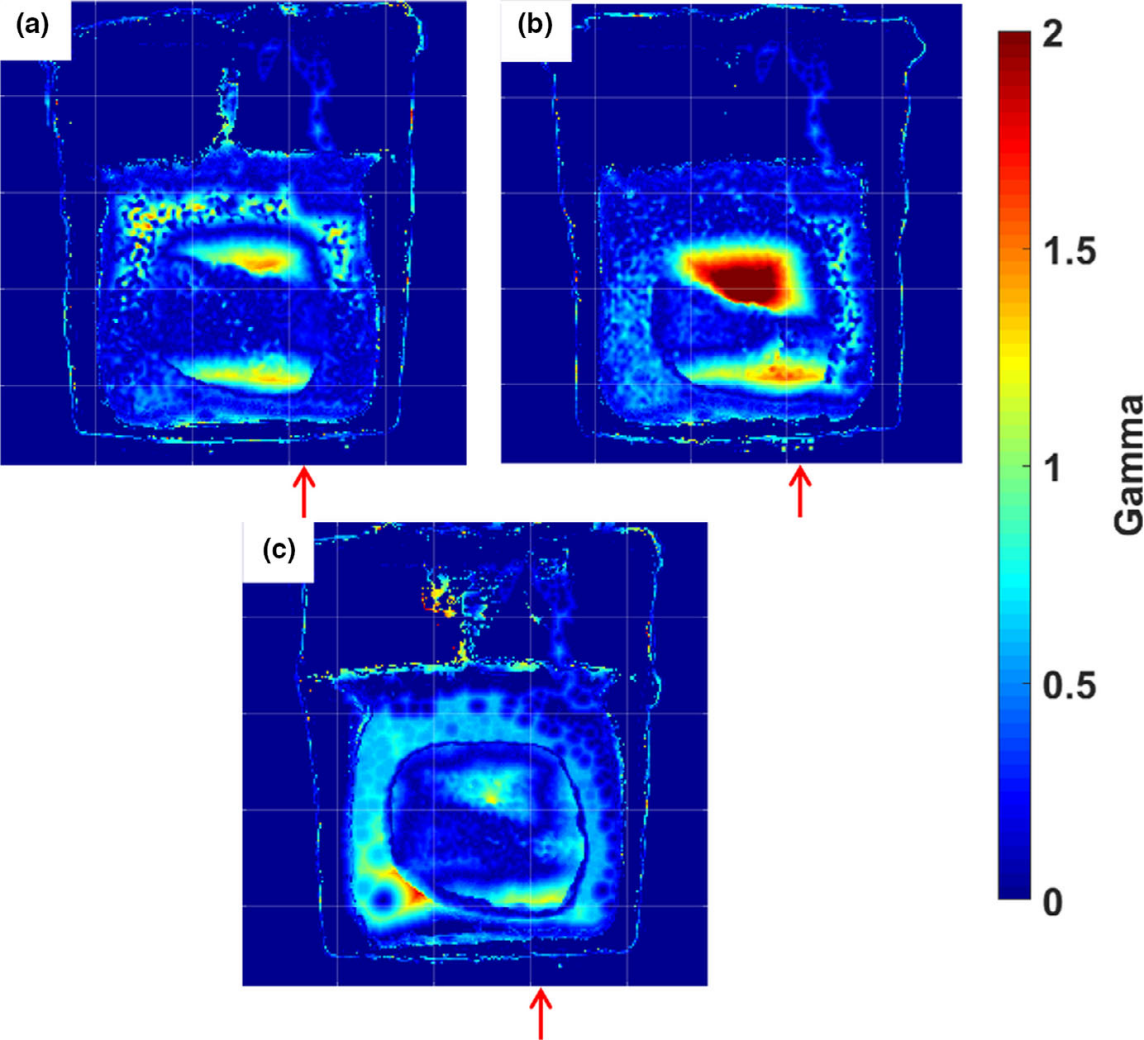
Y‐profiles through each dose distribution measured by the dosimeters during the dynamic deformation experiment.

**Figure 12:**
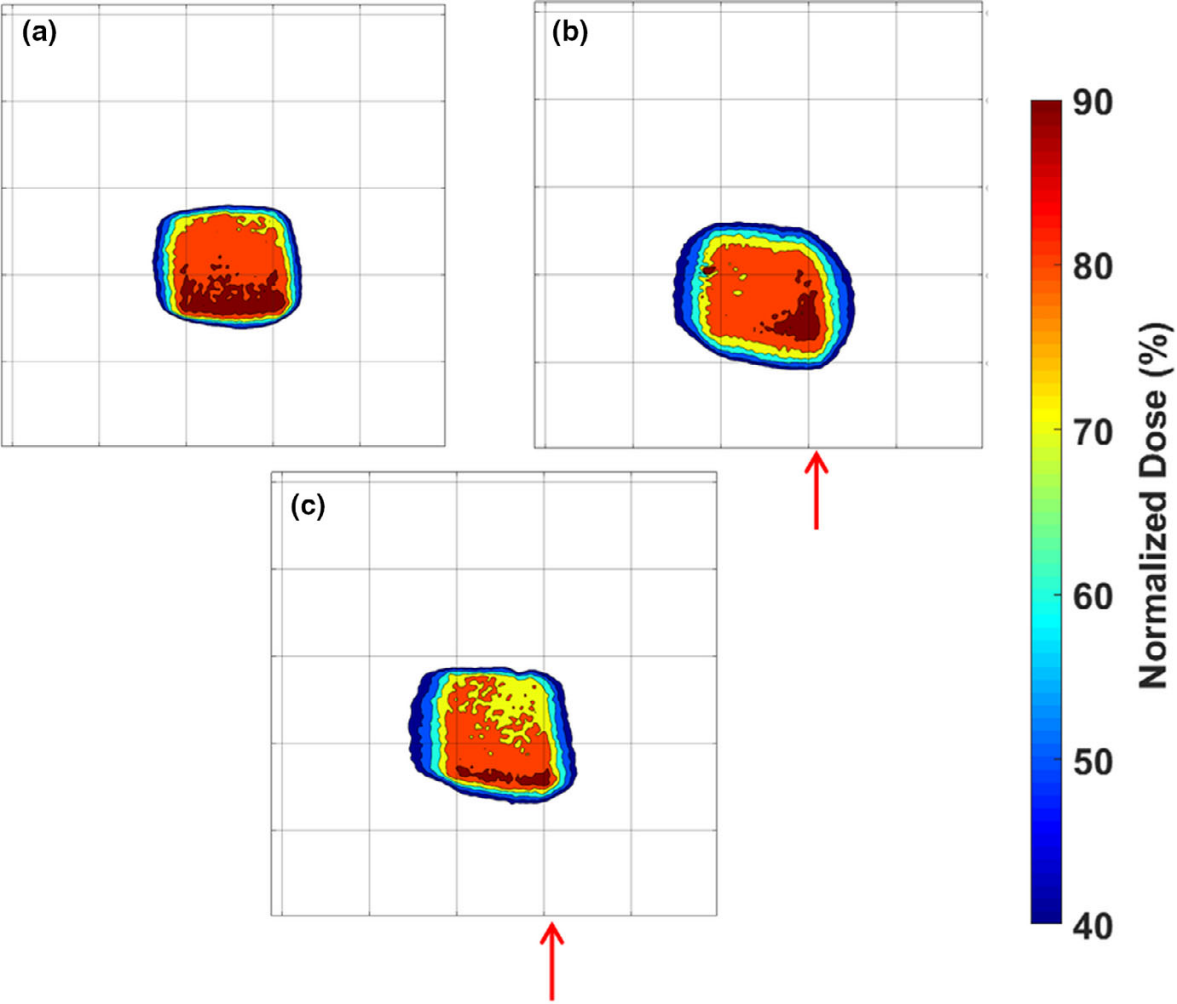
Central slices of γ maps utilizing 3%/5 mm criteria. The dynamically deformed dosimeter without beam gating (a) and the dynamically deformed dosimeter that utilized beam gating (b) were compared to the static undeformed dosimeter. Red arrows approximate the location of the deformation site and the direction of deformation. Each grid mark is spaced by 2.5 cm.

The central coronal slices of the isodose maps of each dosimeter are shown in Fig. 10. Without beam gating, dynamic deformation measurements revealed a shift toward the site of deformation in the bottom right corner of the isodose map and widening and blurring of the high‐dose region. The shift toward the site of deformation was not present when beam gating was used during the treatment. Figure 11 shows an IEC defined y‐profile through each of the dosimeters, which further illustrates the shift of the ungated dosimeter and the blurring measured from both dosimeters. The undeformed dosimeter was compared to the planned dose distribution and was found to have a 96.4% pass rate for 3%/5 mm criteria and a 97.4% pass rate for 5%/5 mm criteria. Example central coronal slices of the γ maps of the comparison of the dynamically deformed dosimeters to the undeformed dosimeter are shown in Fig. 12, and the pass rates are shown in Table 3. γ pass rates were calculated using the same 20% threshold as the static deformation experiment.

**Table 3:** Pass γ pass rates of the comparison of the dynamically deformed dosimeters to the static undeformed dosimeter.

**Dosimeter**	**Dose Difference (%)**	**DTA (mm)**	**Pass Rate (%)**
Ungated	3	5	77.3
Ungated	5	5	79.3
Beam gated	3	5	93.7
Beam gated	5	5	95.2

DTA, distance‐to‐agreemen.

## 4 DISCUSSION

This work presents a deformable abdominal phantom featuring a deformable 3D gel dosimeter developed for the testing of dose accumulation algorithms and real‐time motion management systems. PVCP has many advantages as an anthropomorphic phantom material due to its deformability and high degree of customizability for multimodal imaging. The composition of phantom materials can be modified to enhance contrast in CT and MRI or alter the radiological properties in order to accurately represent specific organs of the body. With the addition of an imaging window in the phantom apparatus and an ultrasonic scatter such as graphite mixed in with the PVCP, the phantom can be used with ultrasound imaging and will have a tissue‐like appearance.^28^ These properties of the PVCP material allow for a more versatile phantom that can aid in the testing of a variety of systems that utilize imaging as a motion management method. Also, the addition of the fully programmable motion‐driving system allows for the use of realistic motion traces during treatment including simple static deformations.

The pairing of a deformable nPAG dosimeter with the deformable phantom apparatus allows for robust testing of different systems using 3D measurements thus permitting visualization of the full effects of motion, as opposed to single‐point or single‐plane measurements. Although previous studies have used deformable 3D dosimeters for the testing of deformable dose algorithms and for real‐time IGRT validation with rigid motion phantoms, to the best of our knowledge, this is the first implementation of deformable 3D dosimetry in a deformable anthropomorphic phantom. This pairing allows for incorporation of both deformation and translation of the dosimeter in three dimensions during treatment, which is more representative of motion in the human body.^41^

Measurements of a liver SBRT treatment in a case without motion or deformation were performed to ensure that in‐phantom measurements match planned dose distributions. γ‐analysis revealed good agreement between the dosimeters and the planned dose in the high‐dose target regions, which demonstrates the ability of these dosimeters to measure the high target doses characteristic of SBRT without experiencing response saturation. Only two regions of the dosimeters showed higher amounts of γ failures. One region was at the edges of the dosimeters, which were primarily caused by oxygen contamination in the nPAG causing an inhibition of the polymerization reaction in the gel dosimeter.^42^ This can be mitigated by keeping the target ROI toward the center of each dosimeter. The other region was the leading and following penumbra regions of the target region in the direction that image slices were gathered during MRI. This was caused by large dose gradients captured within coarser 3 mm thick slices during the MRI scan. This, in turn, caused slight shifts in dose map coregistration or partial volume effects, which exhibited discrepancies between the planned and measured distributions. The high γ pass rates in the central slice of the dose maps also suggest that the majority of discrepancies occurred in the penumbra regions in the slice direction. Therefore, it is important to either choose an MRI slice direction that has a low‐dose gradient, or if this is not possible, choose a slice direction in a direction of lower interest. This also means that one should not select a slice direction in the direction of motion when motion is incorporated. With these guidelines taken into account, the measurements of the nPAG dosimeters placed in‐phantom during irradiation were shown to be reliable and match well with planned dose distributions.

Static deformation created by the motion stage plunger is consistent with shifts toward deformation sites shown by previous work in deformable 3D dosimetry.^22^ In the case of the dosimeters irradiated with a single fraction, the deformation resulted in distinct shifting in the higher isodose levels. Specifically, the plunger pushed the medial side of the dosimeter (the right‐hand side of Fig. 7), resulting in the section of gel closer to the plunger to be pushed into the beam, while a section of the gel further from the plunger was pushed out of the beam. When the gel is returned to its original shape at which the treatment was planned at, this push into and out of the irradiated region appears as a shift of the dose distribution approximately equal to the magnitude of deformation on the cranial edge of the phantom, as shown by the profiles in Fig. 8. The dosimeter irradiated with three fractions showed the effects of multiple deformations over the course of treatment, which caused a blurring of the cumulative dose over a large volume. This blurring can be observed in Fig. 8 that shows a step‐like nature in the profile due to the three distinct deformation states the dosimeter was irradiated in. This case simulates a patient treatment where patient anatomy may show slight day‐to‐day shifts and deformations.

The phantom apparatus showed through these measurements the ability to simulate and directly measure the effects of interfractional variations. This suggests that the apparatus could be used in the future for the comparison to the calculations of the deformable dose accumulation algorithms which monitor these day‐to‐day variations through γ‐analysis and other metrics. During the static deformation measurements, the increase from a 1 cm deformation to a 2 cm deformation resulted in a large shift in the dose distribution, which caused the γ pass rate to decrease, indicating that increasing shifts and deformations can be a detriment on plan delivery quality. The γ‐analyses of the three‐fraction dosimeter showed that the blurring of the dose distribution over the multiple fractions resulted in higher pass rates than the single fraction dosimeter deformed 2 cm, but lower pass rates than the 1 cm deformation single fraction dosimeter. A possible cause of this is the first delivered fraction did not include any deformation, partially decreasing the cumulative dose distribution shift of the deformations during the second and third fraction deliveries. This experiment showed another versatile feature of the phantom apparatus, namely the ability of the phantom to quantify the effects of simple static shifts during a single fraction, along with the cumulative effects of different anatomy shifts and deformations over multiple fractions. This allows for the robust testing of deformable dose accumulation algorithms for calculations over varying fractionation schemes.

The effects of beam gating were investigated using the phantom apparatus. The dynamically deformed dosimeter with an ungated treatment showed a distinct shift toward the site of deformation due to the nature of the motion trace. Since the motion trace began with the dosimeter in the undeformed position used during treatment planning, the high‐dose region of the dosimeter was shifted toward the site of deformation. The dosimeter profile in Fig. 11 also showed some distinct widening of the ungated dynamically deformed dosimeter when compared to the undeformed static case. This was primarily due to the deformation of the dosimeter causing the gel to be slightly compressed during the irradiation. When the dosimeter was imaged in an undeformed state by MRI, the compressed sections of the gel relaxed back to their original positions causing a widening of the high‐dose profile. Although the central position of the dosimeter had its motion accounted for with beam gating, a widening of the high‐dose region was still observed. This was likely due to the fact that the dosimeter was still being dynamically deformed during treatment and had a 30% duty factor for its gating window. While the treatment beam was gated on, the dosimeter was still slightly deformed by the phantom plunger compressing the dosimeter toward the treated region of the dosimeter. The treatment delivery benefits of gating the beam to account for the phantom motion were well illustrated by the phantom. The gated dosimeter showed a distinct decrease in dose distribution shift compared to the ungated dosimeter. Also improvement in the γ pass rates of the gated dosimeter when compared to the dosimeter that did not include beam gating was displayed. The γ maps showed that the gated dosimeter did not have the bands of high γ values characteristic in the ungated dosimeter due to the shift of its dose distribution. During the development of a real‐time motion management system, this would be an important test to ensure that the use of the system actually improves treatment delivery when compared to a case without motion management or a different motion management method. Although this experiment used beam gating based on a known motion trace and not real‐time image guidance, this phantom apparatus could feasibly be modified with the addition of ultrasonic scatterer or a MRI‐compatible motion stage to test ultrasound or MRI‐based real‐time IGRT systems. Additionally, fiducial markers potentially could be implanted within the phantom in the future to be used with EPID‐based or fluoroscopy‐based image guidance systems.

## 5 CONCLUSIONS

A deformable abdominal phantom was developed to contain a removable, deformable nPAG dosimeter to represent a liver tumor within a deformable anthropomorphic phantom. Measurements with the phantom and dosimeter where initially benchmarked with static irradiations measurements that matched well between the dose distributions planned with Eclipse^tm^ and the nPAG dosimeter. The ability to measure the effects of static deformations during treatment and compare these measurements back to a baseline undeformed case was then demonstrated with the phantom. This allows for the phantom apparatus to be used to provide direct measurements of the effects of these deformations and could be used for comparison with calculations made by deformable dose accumulation algorithms in the future. The phantom apparatus was also used to quantify the effects of intrafractional motion and potential improvements of treatment delivery due to the incorporation of beam gating with the intent being to provide a robust test to ensure a system provides a quantifiable benefit to treatment delivery among different real‐time IGRT systems. The phantom apparatus developed during this work shows great potential to provide an excellent method for testing and improving the systems currently being developed to monitor and manage patient motion during radiotherapy and improve treatment delivery.

## CONFLICT OF INTEREST

This work was partially funded by NIH grant R01CA190298. Bryan Bednarz is a Co‐Founder and CSO of Voximetry, LLC, but this did not affect the work presented in this manuscript. None of the other authors have any other conflicts of interest to disclose.
